# *Falsirhodobacter* sp. alg1 Harbors Single Homologs of Endo and Exo-Type Alginate Lyases Efficient for Alginate Depolymerization

**DOI:** 10.1371/journal.pone.0155537

**Published:** 2016-05-13

**Authors:** Tetsushi Mori, Mami Takahashi, Reiji Tanaka, Hideo Miyake, Toshiyuki Shibata, Seinen Chow, Kouichi Kuroda, Mitsuyoshi Ueda, Haruko Takeyama

**Affiliations:** 1 Faculty of Science and Engineering, Waseda University, Tokyo, Japan; 2 Department of Life Sciences, Graduate School of Bioresources, Mie University, Mie, Japan; 3 Research Center for Aquatic Genomics, National Research Institute of Fisheries Science, Kanagawa, Japan; 4 Division of Applied Life Sciences, Graduate School of Agriculture, Kyoto University, Kyoto, Japan; MJP Rohilkhand University, INDIA

## Abstract

Alginate-degrading bacteria play an important role in alginate degradation by harboring highly efficient and unique alginolytic genes. Although the general mechanism for alginate degradation by these bacteria is fairly understood, much is still required to fully exploit them. Here, we report the isolation of a novel strain, *Falsirhodobacter* sp. alg1, the first report for an alginate-degrading bacterium from the family Rhodobacteraceae. Genome sequencing reveals that strain alg1 harbors a primary alginate degradation pathway with only single homologs of an endo- and exo-type alginate lyase, AlyFRA and AlyFRB, which is uncommon among such bacteria. Subsequent functional analysis showed that both enzymes were extremely efficient to depolymerize alginate suggesting evolutionary interests in the acquirement of these enzymes. The exo-type alginate lyase, AlyFRB in particular could depolymerize alginate without producing intermediate products making it a highly efficient enzyme for the production of 4-deoxy-L-erythro-5-hexoseulose uronic acid (DEH). Based on our findings, we believe that the discovery of *Falsirhodobacter* sp. alg1 and its alginolytic genes hints at the potentiality of a more diverse and unique population of alginate-degrading bacteria.

## Introduction

Alginate-degrading bacteria are bacteria that have the ability to degrade the algal polysaccharide, alginate, composed of long heteropolymeric chains of randomly aligned monosaccharides, **β**-D-mannuronate and **α**-L-gluronate. These bacteria harbor unique genes expressing “alginolytic” enzymes comprised of alginate lyases, membrane transporters, reductases and kinases. In general, these microbes secrete alginate lyases that cleave large alginate polymers to oligomeric sugars where membrane transporters transport the oligomers intracellularly. The oligomeric sugars are subsequently depolymerized to unsaturated uronic acids by intracellular alginate lyases and are non-enzymatically converted to the **α**-keto acid, 4-deoxy-L-erythro-5-hexoseulose uronic acid (DEH). With the presence of a series of reductases and kinases, DEH is finally converted to 2-keto-3-deoxy-6-phosphogluconate (KDPG) to be assimilated into the Entner-Dourdoroff pathway [1, 2].

Since the initial discovery of these bacteria [1], alginate-degrading bacteria have been considered as important targets to industry due to their commercial value. Thus, numerous studies have been performed to exploit their alginolytic genes [3, 4]. Among these genes, alginate lyase in particular, is of great importance since the enzyme can depolymerize alginate to its oligomers or monomeric constituents. Extraction and characterization of alginate lyases have led to the classification of endo- and exo-type enzymes where the enzymes or the end products can be applied in pharmaceutics [5, 6], food industry [7] and recently in bioethanol production [8, 9]. As a result, various research groups have pursued the identification and isolation of such alginolytic strains from the environment leading to the discovery of several key strains namely *Sphingomonas* sp. strain A1 [10], *Saccharophagus degradans* strain 2–40 [11], *Zobellia galactanivorans* [12], *Sphingomonas* sp. MJ-3 [13] and *Vibrio splendidus* strain 12B01 [14].

In addition, alginate-degrading bacteria are also considered important in evolutionary research where studies on the transfer of alginolytic genes among bacterial strains have revealed that several of these microbes harbor large alginolytic operons that comprise of the necessary enzymes for alginate degradation [12]. This has facilitated the establishment of metabolically engineered microbes in which these large alginolytic operons could be recruited and introduced into ethanologenic hosts such as *Sphingomonas* sp. strain A1 [15], *Escherichia coli* [8] and even *Saccharomyces cerevisiae* [9]. Currently, alginate-degrading bacteria are mainly distributed into two major phyla, which are Bacteroidetes and Proteobacteria [12].

Although numerous alginate-degrading bacteria and its alginolytic enzymes have been identified thus far, further understanding of alginate-degrading bacteria including evolutionary and bacterial diversity, the efficacy of alginate degradation and detailed functional studies of the enzymes are still required to fully exploit these microbes. Thus, reports on the crystal structures of alginate lyases [16, 17] and continuous reports on the isolation of new strains and enzymes are still ongoing [18]. Furthermore, online databases such as the Carbohydrate-Active enZYmes (CAZy) database hold numerous sequences of alginate lyases that are not yet fully characterized or are of unknown origin suggesting the possibility of elucidating unknown characteristics of the enzyme. In this work, we focused on answering several of these questions by screening various environmental samples to search for novel alginate-degrading bacteria and subsequently characterizing its alginate lyases to search for new and unique features among them.

## Materials and Methods

### Resources

Eight types of brown algae, *Ecklonia cava, Padina arborescens, Sargassum macrocarpum, Dictyota dichotoma, Sargassum ringgoldianum, Sargassum fusiforme, Colpomenia sinuosa, Eisenia bicyclis*, and two gastropods, wedge sea hare (*Dolabella auricularia*) and horned turban (*Turbo cornutus*) were collected at a sublittoral area of Arasaki (Coordinates: 35.197 N, 139.598 E), Kanagawa Prefecture, Japan. The gastropods were individually reared in open-aired aquariums constantly circulated with sand-filtered seawater with water temperatures ranging between 14–22°C at the rearing facility of the Yokosuka Laboratory of the National Research Institute of Aquaculture. Aquarium aeration was conducted using standard aquarium air stones. The gastropods were fed with *E. cava* for four weeks and the feces and gut were collected accordingly.

### Bacterial cultivation and culture medium

All isolated marine bacteria were cultured and maintained in Difco Marine broth 2216 (MB; Difco). Marine broth was filter sterilized prior to use upon autoclaving. Bacterial cultivation was conducted on marine agar or in liquid medium under agitation at 25°C. Glycerol stocks were established using 20% glycerol. *Escherichia coli* strain BL21 was used for cloning and protein expression. *E*. *coli* was cultured with agitation at 37°C in Luria-Bertani broth (LB; Merck).

### Enrichment and isolation of alginate-degrading bacteria

Brown algae were placed in conical flasks filled with 100 mL of seawater and were allowed to ferment at 25°C for 30 days. Feces samples were disrupted using zirconia beads in PBS. Debris from the fermentation and disruption processes was allowed to settle prior to supernatant collection. The supernatant from each treatment was collected and centrifuged at 3,000 g, 10 min to attain the bacterial fraction. Alginate-degrading bacteria were enriched from the collected bacterial fractions by 3 cycles of cultivation in 4X diluted MB supplemented with sodium alginate (Sigma; viscosity ≥ 2000 cP) at 25°C. In each cultivation cycle, alginate concentration was sequentially increased from 0.1% to 0.5% and 1%. Finally, the enriched bacterial cultures were spread and cultured on marine agar plates supplemented with 1% sodium alginate at 30°C for 3 days. Alginate-degrading bacteria were identified by halos surrounding the colonies upon spraying with 95% ethanol, colony morphology and thin layer chromatography (TLC).

### Thin layer chromatography (TLC)

Protein solutions reacted with 1% sodium alginate at a ratio of 1:1 (v/v) were spotted on HPTLC Silica gel 60 F_254_ plates (Millipore) and developed with 1-butanol: acetic acid: dH_2_O (3: 2: 2, v/v). Degraded products were visualized with naphthoresorcinol solution [19] by heating at 135°C for 10 min. Naphthoresorcinol solution was prepared by diluting 1 mg of naphthoresorcinol in 10% sulfuric acid, 50% ethanol and dH_2_O to a total volume of 1 mL. The naphthoresorcinol solution can be scaled up accordingly. Hydrolyzed sodium alginate, degraded alginate products treated with cell extract from *Saccharophagus degradans* and a commercially available alginate lyase from *Flavobacterium multivurom* (Sigma) were used as positive controls. MB and sodium alginate were used as negative controls.

### PCR amplification

All genes in this work were amplified by PCR using the PrimeSTAR Max DNA polymerase (TaKaRaBio) based on the manufacturer’s protocol. 16S rRNA genes from each bacterial isolates were amplified using the 16SU27F (5’—AKWGTTTGATCMTGGCTCAG) and the 16SU1492R (5’—GGHTACCTTGTTACGACTT) eubacterial primer sets. The ORFs, *alyFRA* and *alyFRB*, each encoding for the AlyFRA and AlyFRB alginate lyases were amplified by PCR from the genome of *Falsirhodobacter* sp. alg1 (accession number BBJC02000000) using the respective specific primer sets: alyFRA-F (5’–**CAT****ATG**AGTCTGAAGCTACGCAC) and alyFRA-R (5’–**CTCGAG**GCTGCCGCGCGGCACCAGTTCGCCGTGCGTCAC) for *alyFRA* and alyFRB-F (5’—**CAT****ATG**TCGACGGAAAACAAATCCCGTT) and alyFRB-R (5’—**CTCGAG**GCTGCCGCGCGGCACCAGGTTATTTTTGATGCCAGGTGTTC) for *alyFRB*. Bold sequences designate the *Nde*I and *Xho*I restriction enzyme sites and underlined sequences indicate the target gene. A thrombin site, indicated as non-labeled sequences, was included to the C terminus of each sequence for future native protein studies.

### Plasmid construction and extraction

PCR amplified *alyFRA* and *alyFRB* ORFs were cloned into the pET25b vector at the *Nde*I and *Xho*I sites using standard cloning techniques. Successful cloning of the genes was determined by sequencing of the extracted plasmids. Plasmid extraction was conducted using the Wizard Plus SV Miniprep DNA Purification System (Promega).

### Protein overexpression and purification

pET25b expression plasmids harboring either *alyFRA* or *alyFRB* was newly transformed into *E*.*coli* BL21 cells and spread on LB agar plates supplemented with 50 μg/mL of ampicillin to attain single colonies. A single colony of each transformant was selected and cultured overnight (Preculture) in LB under ampicillin selection. 100 **μ**L of the preculture was inoculated to fresh 5 mL LB and isopropyl **β**-D-1-thiogalactopyranoside (IPTG; 1 mM final concentration) was added when cells were at OD_600_ 0.5–0.6 to induce protein overexpression. Cells were subsequently grown for 20 hours at 16°C. Induced cells were collected by centrifugation and disrupted with 0.1 mm glass beads in a multi-beads shocker (Yasui Kikai) Cell homogenates were harvested by centrifugation at 15,500 g, 10 min and protein overexpression was subsequently determined by SDS Polyacrylamide gel electrophoresis (SDS-PAGE). Proteins were purified using the Ni-NTA protein purification kit (Merck Millipore) and protein concentrations were determined with an e-spect spectrophotometer (BM Equipment). Enzyme activity of overexpressed proteins for alginate degradation was determined by incubation with 1% sodium alginate for 24 h followed by analysis of degraded products by TLC.

### Liquid chromatography-mass spectroscopy (LC/MS) analysis

The separation and detection of DEH was carried out using a LC-MS system with a Shodex IC NI-424 column (4.6 i.d. x 100 mm; Showa Denko). The LC-MS system consisted of a 6120 Quadrupole mass spectrometer (Agilent Technologies), a 1260 Infinity LC (Agilent Technologies) and an OpenLAB Chromatography Data System ChemStation (Agilent Technologies). Ionization method of the sample was conducted using the ESI/APCI multimode analysis in negative mode. The peak of DEH was detected using a mass spectrometer with a selected ion monitoring (SIM) detector. The mass to charge ratio was set to 176 that corresponds to the mass number of DEH. Elution was performed at a flow rate of 0.5 mL/min with 40 mM ammonium formate buffer including 0.1% formic acid. The column oven was set at 40°C. Other experimental conditions for the mass spectrometer were as follows: dry gas, 12.0 L/min; nebulizer, 35 psi; dry temperature, 250°C; vaporizer, 200°C.

### Dinitrosalicylic acid (DNS) assay

20 **μ**L of purified protein solutions reacted with 1% sodium alginate at a ratio of 1:1 (v/v) for 24 h were mixed with equal volumes of DNS reagent [20]. DNS reagent was prepared by diluting 5 mg of 3,5-dinitrosalicylic acid and 16 mg potassium sodium tartrate tetrahydrate in 1 mL of 0.4 M NaOH. The DNS reagent can be scaled up accordingly. Mixtures were boiled at 100°C for 10 min, cooled to room temperature for 2 min and absorbance of generated products were measured at 530 nm. All reactions were conducted using 1 **μ**g/**μ**L (final concentration) of purified protein.

### Enzyme characterization and activity assay

Enzyme properties of AlyFRB were determined based on its susceptibility to cationic ions, temperature, alginate depolymerization time and pH by TLC or DNS assay. For the evaluation of cationic ions, purified AlyFRB was assessed with 1.0% sodium alginate in the presence of 30 mM of MgCl_2_, 160 mM NaCl or 3.5 mM KCl in 50 mM MOPS buffer (pH 7.0). Evaluation in MOPS buffer only was used as a reference. The optimal temperature of AlyFRB was measured in MOPS buffer at various temperatures ranging from 25–45°C. The time required for alginate depolymerization was measured at several time points (0, 1, 2, 4, 8 and 16 h) over a 16 h reaction in MOPS buffer at 30°C. The pH optimum of the purified enzyme was determined using the following buffers: 50 mM sodium citrate (pH 3.0 to 6.0), 50 mM MOPS (pH 6.0–8.0), 50 mM HEPES (pH 7.0–8.0) and 50 mM Tris-glycine-NaOH (pH9.0 to 10.0) as previously described [21] at 30°C, 2 h. All reactions were conducted using 40 **μ**g of purified AlyFRB in 40 **μ**L total reaction volumes.

### Genome sequencing and assembly

Genome DNA was extracted from overnight grown *Falsirhodobacter* sp alg1 using the UltraClean Microbial DNA Isolation Kit (MoBio) based on the manufacturers protocol. DNA library for sequencing was prepared using the Nextera DNA Library Prep Kit (Illumina) as recommended and the library was sequenced using an Illumina MiSeq sequencing system accordingly. Quality and concentration of all samples were determined using the 2100 Bioanalyzer (Agilent Technologies). Obtained sequencing reads were assembled using SPAdes excluding the single-cell feature [22]. Contigs attained from the MiSeq sequencing run were integrated with formerly sequenced 454 reads [23] and reassembled using Geneious R8 v. 8.1.5 (Biomatters). Automatic gene annotation and coding sequence classification was performed against the draft genome by Rapid Annotation using Subsystem Technology (RAST) [24]. Operon and standalone genes were predicted using the FGENESB online prediction tool (Softberry) [25]. Genome completeness was confirmed by the identification of conserved single copy genes (CSCGs) [26].

### Identification of alginate lyase genes and data analysis

Comparative analysis of the 16S rRNA gene of bacterial isolates was performed by BLAST against the NCBI database. Oligoalginate lyase sequences used as reference for amino acid sequence comparison were manually extracted from the NCBI database based on sequences from published work. Alginolytic genes related to alginate assimilation of *Falsirhodobacter* sp. alg1 were predicted and identified from the draft genome revised in this work. Classification of alginate lyases were conducted based on the Carbohydrate-Active enZYmes (CAZy) Database. Sequence alignments and phylogenetic trees were generated using Geneious R8 v. 8.1.5. All graphs were prepared using Prism 6.0 (GraphPad). Conserved domains of alginate lyases were determined by the Conserved Domain Search Service of NCBI [27]. Presence of signal peptides were determined using SignalP 4.1 [28].

### Nucleotide accession numbers

*alyFRA* and *alyFRB* has been deposited at DDBJ/EMBL/GenBank under the accession numbers LC081341 and LC081342. The draft genome of *Falsirhodobacter* sp. alg1 was updated and the second version was been reassigned the accession number, BBJC02000000.

## Results

### Isolation of a novel alginate-degrading bacterium, *Falsirhodobacter* sp. alg1, from the Rhodobacteraceae family

**Fig 1:**
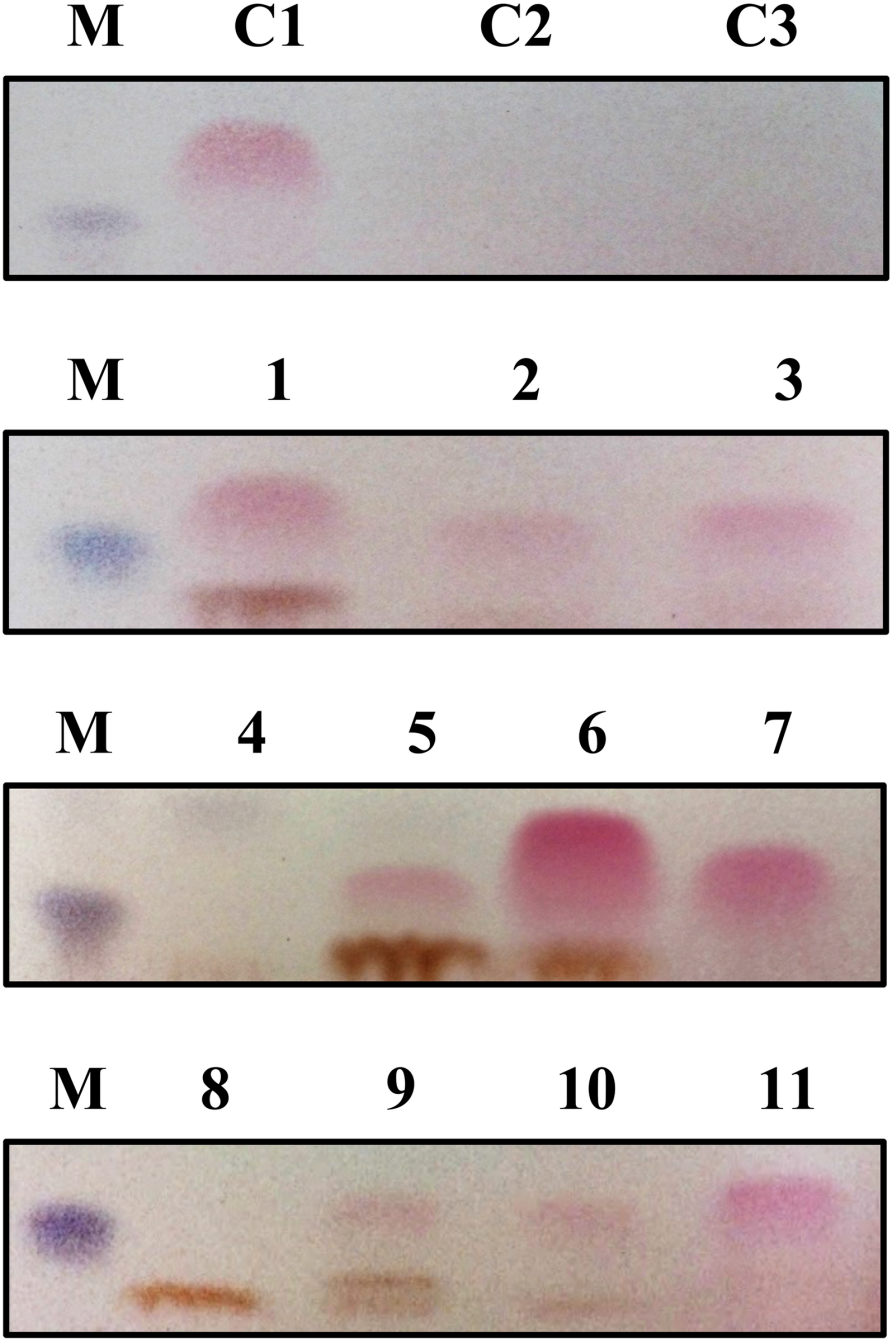
Thin-layer chromatography (TLC) of alginate degraded products using the bacterial strains isolated in this work. The region indicating the presence of alginate monosaccharides are shown since the presence of monomeric sugars indicates the ability of the strains to degrade alginate. M: Hydrolyzed sodium alginate, C1: *Saccharophagus degradans*, C2: Commercial sodium alginate, C3: Marine broth, 1: ADB-BSW-1, 2: ADB-BSW-2, 3: ADB-BSW-3, 4: ADB-BSW-4, 5: ADB-BSW-5, 6: ADB-BSW-6, 7: ADB-BSW-7, 8: ADB-BSW-8, 9: ADB-BSW-9, 10: ADB-BSW-10, 11: ADB-BSW-11. Full TLC images of the degraded products by each strain are provided in S1 Fig in the Supporting Information section for further reference.

From our culture enrichment attempt and upon subjection to alginate assimilation, we successfully attained 11 morphologically distinct bacterial colonies showing strong alginate degrading characteristics *via* the formation of halos. These isolates were subsequently cultured and subjected to TLC to determine their ability to degrade alginate to its monomeric sugars. We found that 9 out of 11 of the isolates could actually degrade alginate to its monomeric sugars (Fig 1). Based on 16S rRNA gene diversity analysis, the bacterial strains isolated showed high similarity, ranging between 96.6%–99.5%, to bacteria from known alginate-degrading bacterial genera such as *Vibrio*, *Pseudoalteromonas*, *Cobetia*, *Formosa*, and *Shewanella* (Table 1). The isolate ADB-BSW-1 was classified as a Bacteroidetes strain while the remaining isolates were classified as Proteobacteria (S2 Fig). Among these isolates, we identified a bacterial strain, ADB-BSW-11, that showed a pairwise similarity of 96.6% to a recently reported novel genus, *Falsirhodobacter*, from the family Rhodobacteraceae [29]. This strain was subsequently termed *Falsirhodobacter* sp. alg1.

**Table 1:** 16S rRNA gene sequence similarity of bacterial isolates with database strains.

Bacterial isolate no.	Resource	Highest similarity	
		Bacterial strain (Accession number)*	Pairwise Identity (%)**
ADB-BSW-1	5	*Formosa algae* strain F89 (NR_029076)	97.9
ADB-BSW-2	1, 2, 5, 6, 7, 8, 9, 11	*Shewanella oneidensis* (AB447987)	99.3
ADB-BSW-3	10	*Shewanella* sp. P1 (JQ867500)	99.1
ADB-BSW-4	1, 4	*Vibrio* sp. EF3B-B166 (KC545338)	99.3
ADB-BSW-5	3	*Vibrio crassostreae* strain LGP 7 (NR_044078)	98.2
ADB-BSW-6	6	*Vibrio* sp. HE1 (FN554590)	98.8
ADB-BSW-7	8	*Vibrio* sp. enrichment culture clone KWE30-5 (JQ670709)	99.2
ADB-BSW-8	5, 9, 10, 12	*Cobetia marina* strain DSM 4741 (NR_042065)	99.7
ADB-BSW-9	8	*Pseudoalteromonas* sp. MMM18 (AY187028)	99.5
ADB-BSW-10	11	*Rheinheimera* sp. 3006 (AM110966)	99.1
ADB-BSW-11	12	*Falsirhodobacter haloterans* strain JA744 (NR_108884)***	96.6

Resource list—1: Feces of Turbo cornutus, 2: Gut extract of Turbo cornutus, 3: Feces of Dolabella auricularia, 4: Gut extract of Dolabella auricularia, 5: Ecklonia cava, 6: Padina arborescens, 7: Sargassum macrocarpum, 8: Dictyota dichotoma, 9: Sargassum fusiforme, 10: Sargassum ringgoldianum, 11: Colpomenia sinuosa, 12: Eisenia bicyclis.

* Only bacterial references with descriptive organism information were selected as the highest similarity bacterial strain, to provide a better representation on the classifications of the bacterial isolates.

** Pairwise identity between reference strains and isolates were determined by Geneious R8 v. 8.1.5.

*** *Falsirhodobacter halotolerans* strain JA744 was designated as the reference sequences based on our previous report [23]

### Gene/operons within *Falsirhodobacter* sp. alg1 related to alginate degradation

In a recent report, we announced the draft genome of *Falsirhodobacter* sp. alg1 [23]. Using these genomic data along with the resequenced genomic data attained from an Illumina Miseq sequencing run conducted in this work, the draft genome was further refined to attain a total genome size of 2,921,973 bp with an average GC contents of 60.2% comprised of 154 contigs (N_50_, 385,563) and 2 circular plasmids sized at 177,157 bp and 10,355 bp. The genome was revised to contain 2,935 coding sequences (CDS) where 1,618 CDS (56%) were classified into 410 subsystems, while 1,317 CDS (44%) were uncategorized. In addition, a total of 138 out of 140 CSCGs were identified from our assembled genome.

**Fig 2:**
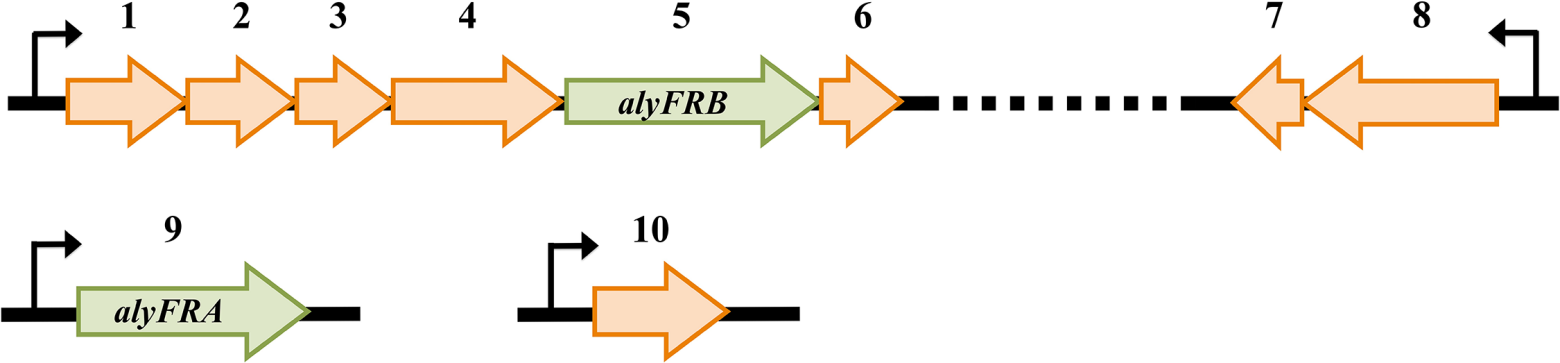
Validated and predicted alginolytic genes/operons within *Falsirhodobacter* sp. alg1. Genes include the following: 1, ATP binding protein UgpC; 2, ABC-type polysaccharide transport system; 3, ABC transporter permease CDS; 4, ABC transporter, substrate binding protein; 5, oligo alginate lyase CDS (*alyFRB*); 6, acetoin (diacetyl) reductase CDS (DehR-like); 7, 4-hydroxy-2-oxoglutarate aldolase (KdpgA-like); 8, phosphogluconate dehydratase; 9, alginate lyase precursor CDS (*alyFRA*); 10, 2-dehydro-3-deoxygluconate kinase (KdgK-like). Orange arrows and green arrows indicate predicted and validated genes respectively. Dotted lines indicate that the operon with the *alyFRB* gene and the operon with the KdpgA-like aldolase were located on the same contig with approximately 43 kbp nucleotides apart. Operons and standalone genes were determined by the FGENESB prediction tool.

Based on this new genome assembly, detailed *in silico* analysis showed that this bacterium had the highest similarity to *Rhodobacter sphaeroides* and harbored all the predicted genes required for the assimilation of alginate (Fig 2). The ORFs coding for two alginate lyases, termed AlyFRA and AlyFRB, were located on separate operons where *alyFRA* was identified as a standalone gene while *alyFRB* was found located on an operon comprised of ABC-type polysaccharide and substrate protein transporters, a permease, an ATP binding protein and an acetoin (diacetyl) reductase (DehR-like). A gene encoding for a 2-dehydro-3-deoxyphosphogluconate aldolase (KdpgA-like) was identified on an operon along with a phosphogluconate dehydratase located approximately 43 kbp downstream to the operon harboring *alyFRB*, while a gene encoding a 2-dehydro-3-deoxygluconate kinase (KdgK-like) was identified as a standalone gene located on a separate contig.

### Efficient degradation of alginate by AlyFRA and AlyFRB

**Fig 3:**
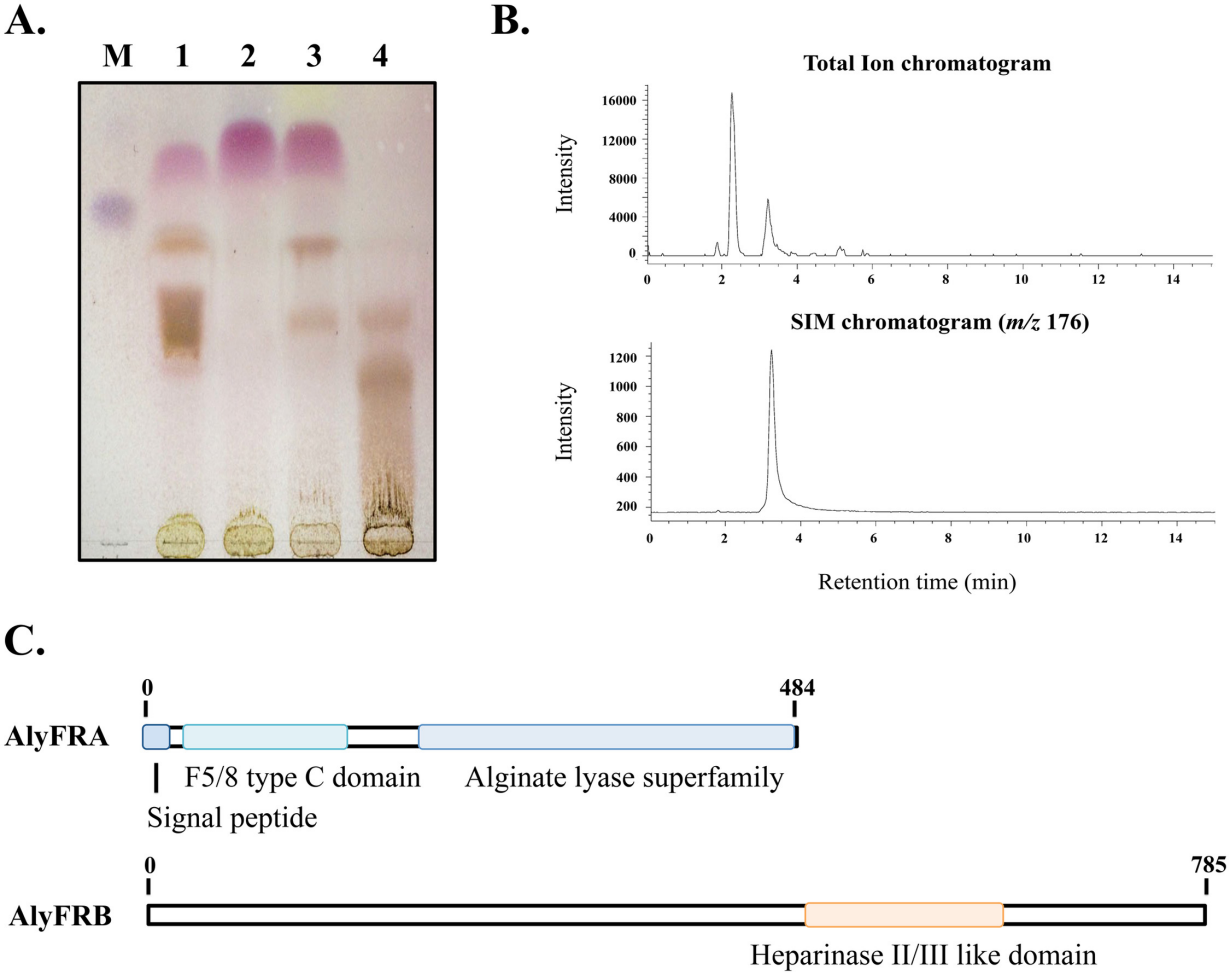
Alginate degrading features of AlyFRA and AlyFRB. a. TLC analysis of alginate-degraded products upon treatment with the respective enzymes. M: Hydrolyzed sodium alginate, 1:AlyFRA, 2:AlyFRB, 3: Alg17C (*Saccharophagus degradans*), 4: Commercial alginate lyase. b. TIC and SIM chromatograms for the identification of DEH within the degraded products of AlyFRB. Peaks from both the TIC and SIM chromatograms at the retention time of 3.2 min indicates DEH. c. Conserved domains identified within AlyFRA and AlyFRB. The conserved domains were determined by using the NCBI Conserved Domain Search software.

Proven that *Falsirhodobacter* sp. alg1 has the ability to degrade alginate to its monomeric sugars, we further investigated the role and characteristics of the alginate lyases AlyFRA and AlyFRB. Both AlyFRA and AlyFRB showed 66.5% protein similarity to an alginate lyase precursor (WP_021695269) and 71.3% to an oligoalginate lyase (WP_021695273) respectively, from *Loktanella cinnabarina* of the family Rhodobacteraceae. We subsequently determined the efficacy of AlyFRA and AlyFRB in alginate depolymerization by TLC and LC/MS analyses. From the TLC analysis, upon confirmation of the overexpression of both AlyFRA and AlyFRB by SDS-PAGE (S3 Fig), AlyFRA and AlyFRB treated with 1% sodium alginate showed that both proteins depolymerized alginate with AlyFRA having endo-like while AlyFRB had exo-like properties (Fig 3A). Interestingly, we found that AlyFRB, although showing exo-like properties, could depolymerize large alginate polymers to its monomeric residues providing a single prominent monomeric band in comparison to AlyFRA and the designated controls. The total ion chromatogram (TIC) from the analysis of AlyFRB degraded products using LC/MS showed the presence of monomeric sugars which was subsequently confirmed to be DEH by SIM based on the mass number (Fig 3B). The presence of DEH was also confirmed by the decrease in *A*_235_ upon alginate degradation, due to the nonenzymatic conversion of unsaturated monosaccharides to the more stable 5-keto structure that does not absorb at 235 nm [1]. To correlate our findings on the properties of AlyFRA and AlyFRB with alginate lyases available in the CAZy database, conserved domain search against both lyases were conducted. We showed that AlyFRA was classified to the PL-7 family harboring a signal peptide, a F5/8 type C domain (E-value: 3.90e-17) and an alginate lyase superfamily domain (E-value: 1.94e-62). AlyFRB on the other hand was classified to the PL-15 family and harbors a conserved Heparinase II/III like domain (E-value: 1.93e-62) (Fig 3C).

### Enzymatic characteristics of AlyFRB

**Fig 4:**
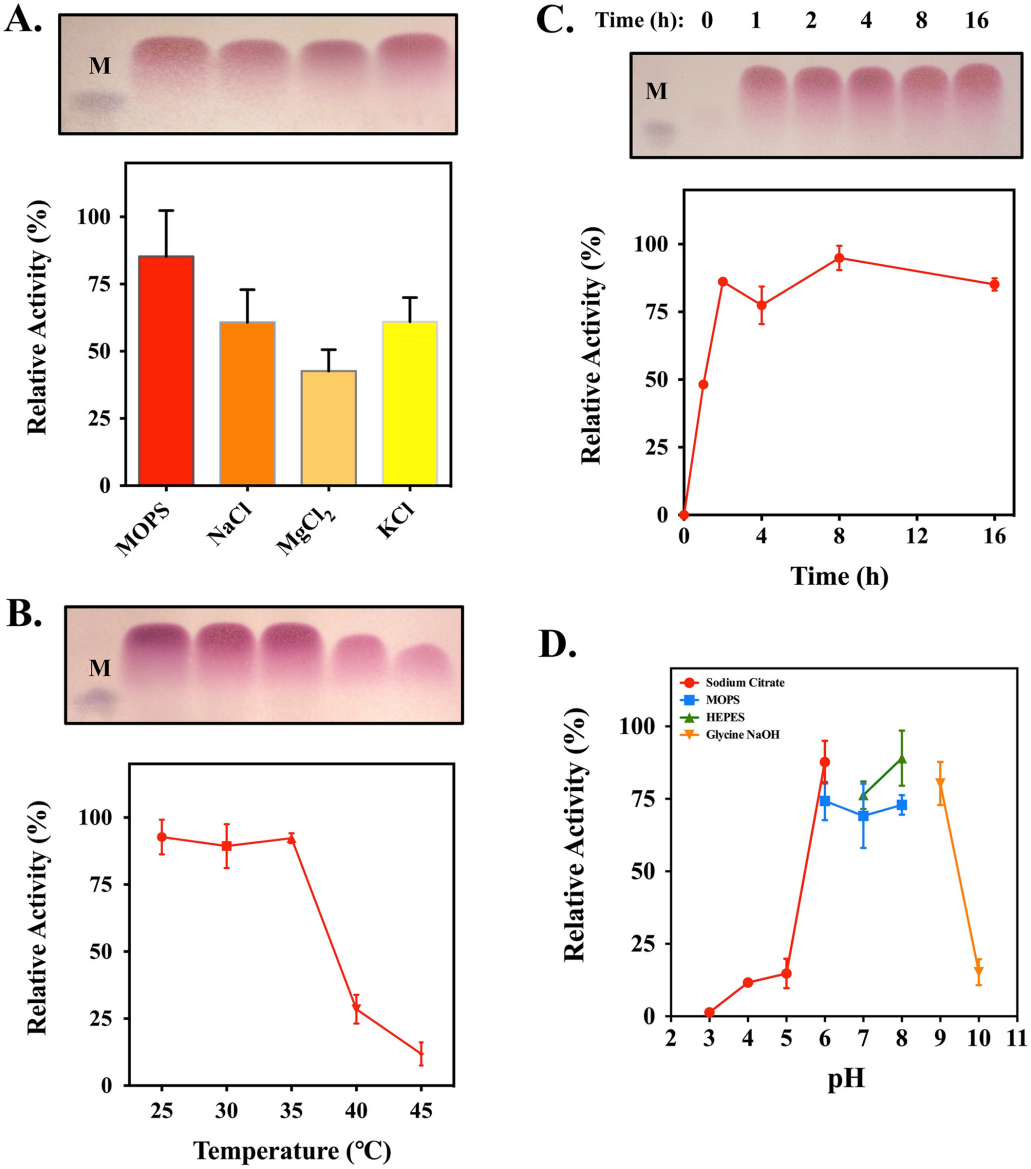
Enzymatic characterization of AlyFRB based on its activity in response to the respective A. cationic ions, B. temperature, C. alginate depolymerization time and D. pH, using TLC and DNS assay. In the analysis of cationic ions, the effects of Na^+^, Mg^2+^, K^+^ ions were evaluated. All reactions were performed in 50 mM MOPS buffer, pH 7.0 with 1 μg/μL (final concentration) of purified protein. Optimal pH analysis was performed at 30°C. Full TLC images of the degraded products for the evaluation with cationic ions, temperature and alginate depolymerization time are provided in S4 Fig in the Supporting Information section for further reference.

To determine the optimal conditions for AlyFRB and to optimize enzyme properties in subsequent applications, evaluation of cationic ions, reactive temperature, alginate depolymerization time and optimal pH was conducted. The data associated to these conditions are shown in Fig 4. To evaluate whether cationic ions are necessary for enzymatic function, AlyFRB tested with Na^+^, Mg^2+^ and K^+^ ions did not show any increase in activity compared to the enzyme in MOPS buffer only (Fig 4A). The optimal temperature for AlyFRB activity was shown to be between 25–35°C (Fig 4B) while the time required for AlyFRB at the designated protein concentration to depolymerize alginate was observed at approximately 1 h after incubation (Fig 4C). No significant increase in alginate depolymerization was observed after 2 h. The optimal pH for AlyFRB was found to lie within pH 6.0–9.0 accordingly (Fig 4D).

### Comparative analysis of AlyFRB with similar homologs

**Fig 5:**
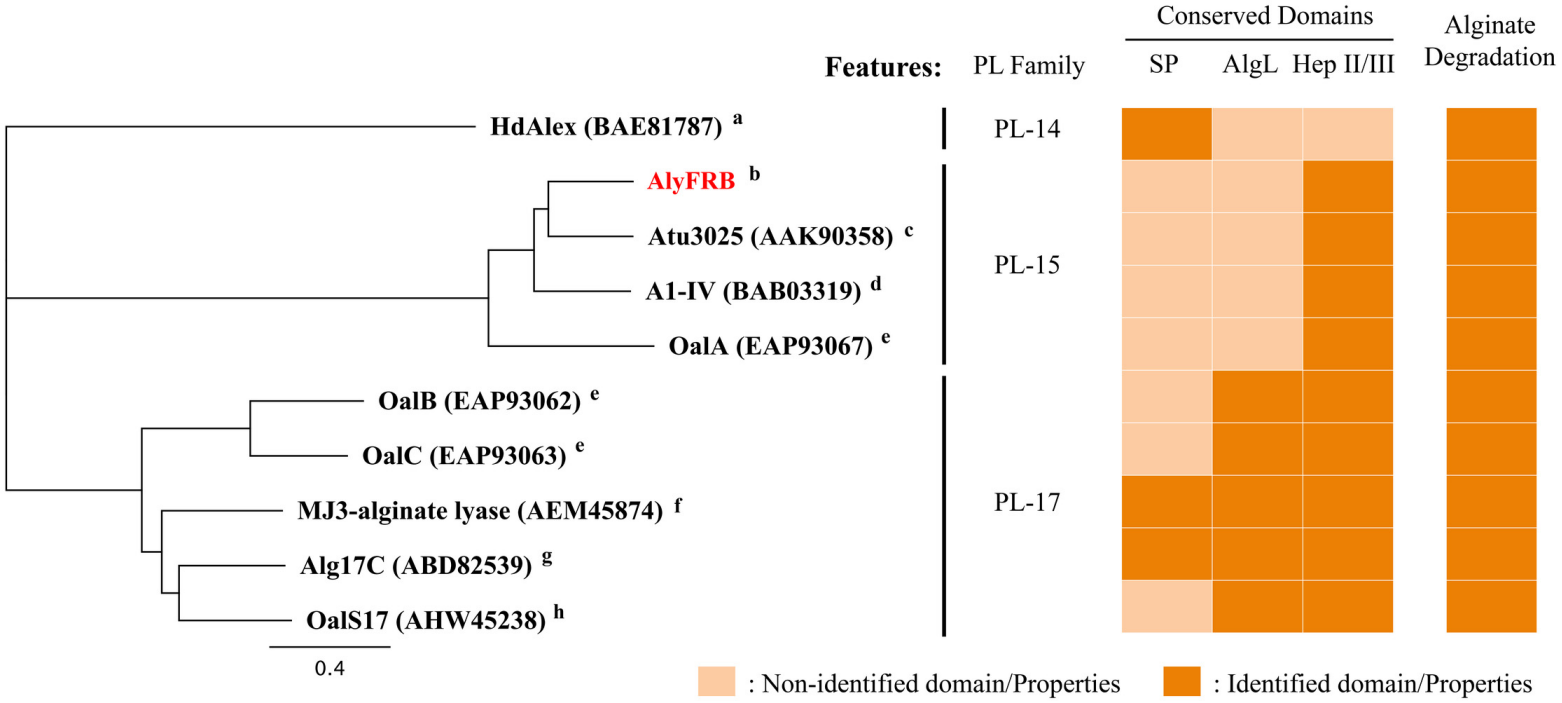
Phylogenetic analysis of currently reported oligoalginate lyases with AlyFRB. Accession numbers of each enzyme are shown in parentheses. Alphabets describe the host origin of the oligoalginate lyase. ^a^ *Haliotis discus hannai* (abalone), ^b^ *Falsirhodobacter* sp. alg1, ^c^ *Agrobacterium fabrum* str. C58, ^d^ *Sphingomonas* sp. strain A1, ^e^ *Vibrio splendidus* 12B01,^f^ *Sphingomonas* sp. MJ-3, ^g^ *Saccharophagus degradans* 2–40, ^h^ *Shewanella* sp. Kz7. HdAlex was assigned as the outgroup.

The phylogenetic representation of AlyFRB with currently reported oligoalginate lyases from the phylum Proteobacteria are described. AlyFRB was clustered with the PL-15 oligoalginate lyases Atu3025 from *Agrobacterium fabrum* (previously termed as *A*. *tumefaciens*) strain C58, A1-IV from *Sphingomonas* sp. strain A1 and OalA from *Vibrio splendidus* strain 12B01 while the lyases from the PL-17 were grouped in a separate cluster (Fig 5). AlyFRB showed the highest amino acid sequence similarity of 58% to Atu3025. As shown, all the lyases harbor the Heparinase II/III like domain while only those from the PL-17 family have the conserved AlgL domain. In addition, several of these enzymes from the PL-17 family showed the presence of signal peptides, which is relatively more common in endolytic alginate lyases. All of these oligoalginate lyases are reported to have the ability to depolymerize large alginate polymers.

## Discussion

### *Falsirhodobacter* sp. alg1 among other alginate-degrading bacteria

As introduced, alginate-degrading bacteria are currently classified into two major phyla, Bacteroidetes and Proteobacteria, in which recent in-depth phylogenomic analyses revealed that these bacteria acquired the alginolytic genes or operons through independent cross-lateral gene transfers from an ancestral marine flavobacterium [12]. Nevertheless, little is still known on the evolutionary diversity of alginate bacteria among other bacterial genera or phyla. In this work, in our effort to search for highly efficient alginate-degrading bacteria, we stumbled upon a novel bacterial isolate, ADB-BSW-11, showing the ability to degrade alginate to its monomeric sugars (Fig 1) and high similarity to the genus *Rhodobacter* within the family Rhodobacteraceae, among our isolated strains (Table 1; S2 Fig). However, since this isolate did not show the common photosynthetic characteristic of *Rhodobacter* strains, we termed it as *Falsirhodobacter* sp. alg1 [23]. *Falsirhodobacter* is a new genus that was recently introduced for bacterial strains that show genetic similarities to *Rhodobacter* but do not show any photosynthetic features [29, 30]. As far as we know, there are no current reports on alginate-degrading bacteria from the family Rhodobacteraceae making strain alg1 as the first to be reported from this family.

To determine the evolutionary status of *Falsirhodobacter* sp. alg1 among alginate-degrading bacteria, we referred to the draft genome of this strain. As previously reported, based on the initial draft genome provided, we briefly described that *Falsirhodobacter* sp. alg1 harbored two alginate lyases, an alginate lyase precursor (AlgFR1; renamed to AlyFRA) and an oligo alginate lyase (AlgFR2; renamed to AlyFRB), and similar genes found within the alginolytic clusters of other alginate-degrading bacteria [23]. However, we were still uncertain on the coverage of the draft genome. One of the main reasons was because identification of single homologs of alginate lyases from each type, endo- and exo-, from alginate-degrading bacteria, to our knowledge, is rare. Majority of the alginate-degrading bacteria currently reported are known to harbor several alginate lyase homologs of each type [11, 14, 21] where the presence of several homologs logically allows bacteria to sequentially and efficiently degrade a target substrate. The only report we know regarding a bacterium harboring only a single alginate lyase was on the *A*. *fabrum* strain C58, harboring the oligoalginate lyase, Atu3025 [31]. Thus, to reconfirm our claims on the possibility of strain alg1 to harbor only single homologs of each alginate lyase type, we resequenced the genome, integrated the sequencing data to the initial draft genome, validated the genome coverage and performed a genome wide search for alginate lyase and related alginolytic genes.

The additional MiSeq sequencing data we integrated to the initial draft genome data, comprised of a total of 2,814,601 bp from 50 assembled contigs and after data integration, we attained a draft genome of approximately 3 Mbp in size. As a result, the addition of the MiSeq sequencing data did not drastically increase the size of the initial draft genome [23]. We subsequently validated a total of 140 CSCGs using a validation method introduced by Rinke et al. [26] and identified 138 CSCGs further showing that the newly assembled genome was near complete. Based on this newly assembled genome, we therefore confirmed that *Falsirhodobacter* sp. alg1 does only harbor single homologs of endo- and exo- type alginate lyases.

We then performed a genome wide search for the alginolytic pathway within this strain to evaluate the distribution of alginolytic genes related to the alginate degradation pathway. Similar to its other Proteobacteria counterparts (with exception to *Vibrio*) [12], *Falsirhodobacter* sp. alg1 lacks the presence of long intact alginolytic operons but instead the alginolytic genes were either found as short operons or standalone genes, arbitrarily distributed within its genome. Having identified all the possible gene candidates related to alginate degradation, we found that strain alg1 harbors a single primary alginolytic pathway for alginate degradation (Fig 2). Here, we hypothesized that the presence of single homologs of each alginate lyase type and a primary alginolytic degradation pathway could possibly indicate that this bacterium may have acquired the necessary alginolytic genes from closer descendants or it may have undergone a selective process to keep the most efficient enzyme within its genome.

### Efficient alginate lyases from *Falsirhodobacter* sp. alg1

Having determined the possibility of strain alg1 to harbor only single homologs of each alginate lyase type, we focused on the degradation efficacy of the enzymes, AlyFRA and AlyFRB. Based on our protein expression results (Fig 3A), we showed that AlyFRA and AlyFRB each degraded alginate in an endolytic and exolytic manner correlating to their PL classification and protein similarity analyses to currently reported alginate lyases. AlyFRA harbored conserved regions typical to endolytic lyases and the presence of a signal peptide suggests that this lyase may function as the initial enzyme for the degradation of large alginate polymers prior to cellular uptake (Fig 3C). AlyFRB, in particular was intriguing since it showed properties to strongly depolymerize alginate to its monomeric constituents (Fig 3A and 3B). In fact, our LC/MS analysis against the depolymerized products of AlyFRB reacted with sodium alginate clearly showed that no dimers, trimers or other oligomers, but only monomeric sugars in the form of DEH could be detected. This result speculates that AlyFRB may be an extremely strong exolytic alginate lyase with the ability to possibly depolymerize large alginate polymers. The ability of both AlyFRA and AlyFRB to efficiently depolymerize alginate could explain the evolutionary relevance of this bacterium and the irrelevance for it to harbor additional homologs of each enzyme type.

Subsequently, further intrigued by the efficiency of AlyFRB in alginate depolymerization, we conducted a search on currently reported alginate lyases and found several enzymes showing similar alginate depolymerization characteristics to AlyFRB [13, 14, 18, 31–35]. Among these enzymes, the most prominent examples showing strong affinity to alginate polysaccharides include A1-IV from *Sphingomonas* sp. strain A1 [33], Atu3025 from *A*. *fabrum* strain C58 [16], Alg17C from *S*. *degradans* [17, 35] and MJ3 from *Sphingomonas* sp. strain MJ-3 [13]. Interestingly, from our phylogenetic analysis of currently reported oligoalginate lyases (Fig 5), we found that AlyFRB showed the highest similarity to Atu3025, which was the oligoalginate, identified as a single homolog within *A*. *fabrum*. Furthermore, when we looked at the orientation of the genes within the operon with the *alyFRB* gene from strain alg1 and that with the gene cluster with the *atu3025* gene from *A*. *fabrum*, they were highly conserved (S5 Fig). In a previous report, Ochiai et al. showed that a similar comparison was conducted between the gene cluster with the *atu3025* gene from *A*. *fabrum* with the *Sphingomonas* sp. strain A1 and concluded that both the strains may have derived from a common ancestor [16]. From our phylogenetic analysis, we also found that AlyFRB showed similarity (51%) to the alginate depolymerization efficient A1-IV oligoalginate from *Sphingomonas* sp. strain A1 (Fig 5). Thus, we can also speculate that the alginolytic gene cluster with the *alyFRB* gene from strain alg1 could have derived from the same ancestor with *Sphingomonas* sp. strain A1 and *A*. *fabrum*. Based on this observation, as far as we know, we think we see for the first time in the context of alginolytic gene clusters, an evolutionary link between bacterial strains with high numbers of alginate lyases such as *Sphingomonas* sp. strain A1 with those with low numbers of alginate lyases such as *A*. *fabrum* and our *Falsirhodobacter* sp. alg1 strain.

Whether this conserved alginolytic gene cluster between the strains was transferred from bacteria with higher number of gene homologs to the lesser one or whether the transfer is bilateral still needs to be determined. Furthermore, the evidence of all 3 strains to have a conserved alginolytic gene cluster with an oligoalginate showing efficient alginate depolymerization characteristics may not be a coincidence and could suggest a selective transfer of efficient enzymes between the strains.

## Conclusion

In summary, upon the discovery of the novel bacterium *Falsirhodobacter* sp. alg1 and analyzing the genome and the efficacy of its alginate lyases in alginate depolymerization, we believe that we have hinted the potentiality for alginate-degrading bacteria to be more diverse and unique. In addition, we also showed for the first time a possible evolutionary link between alginate-degrading bacteria with high and low numbers of enzymes and have hinted on the possibility of the lateral gene transfer of efficient genes between them. Although a full comparative study of AlyFRA and AlyFRB in alginate depolymerization with alginate lyases of similar characteristics was not conducted, we feel that the efficiency of both the enzymes as presented in this work can also serve as an important point for the necessity and the potentiality for new and novel alginate-degrading bacterial strains to be discovered.

## Supporting Information

S1 Fig TLC of alginate degraded products using the bacterial strains isolated in this work (Original figures). Description of lanes is provided in Fig 1 of the main text.
(PDF)

S2 Fig 16S rRNA gene phylogenetic comparison of bacterial strains isolated with database strains. Isolates are indicated in red. Key alginolytic strains *Zobellia galactanivorans* strain DsiJ, *Sphingomonas* sp. A1 and *Saccharophagus degradans* strain 2–40 were used as reference. *Aquifex pyrophilus* strain Kol5a was used as the outgroup. Phylogenetic tree was generated using Geneious R8 v. 8.1.5.
(PDF)

S3 Fig SDS-PAGE gel image of purified AlyFRA and AlyFRB. Samples were separated in a 12% Mini-PROTEAN TGX Stain-Free Protein Gel (Biorad) and visualized with a standard UV transilluminator.
(PDF)

S4 Fig TLC for the enzymatic characterization of AlyFRB (Original figures). Enzymatic activity of AlyFRB in response to a. cationic ions, b. temperature and c. alginate depolymerization time.
(PDF)

S5 Fig Alginolytic gene cluster comparison of *Falsirhodobacter* sp. alg1 and *A*. *fabrum* str. C58. Multiple sequence alignment was generated using Geneious R8 v. 8.1.5.
(PDF)
