# Supplementary figures and images for: Falsirhodobacter sp. alg1 Harbors Single Homologs of Endo and Exo-Type Alginate Lyases Efficient for Alginate Depolymerization
Source: PLoS One. 2016 May 13;11(5):e0155537. doi: 10.1371/journal.pone.0155537 (PMC4866713; doi:10.1371/journal.pone.0155537)

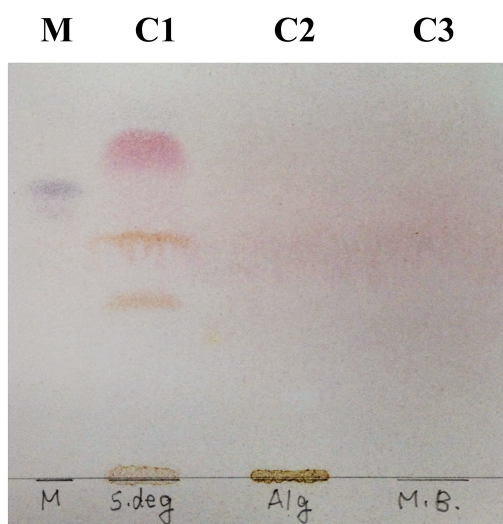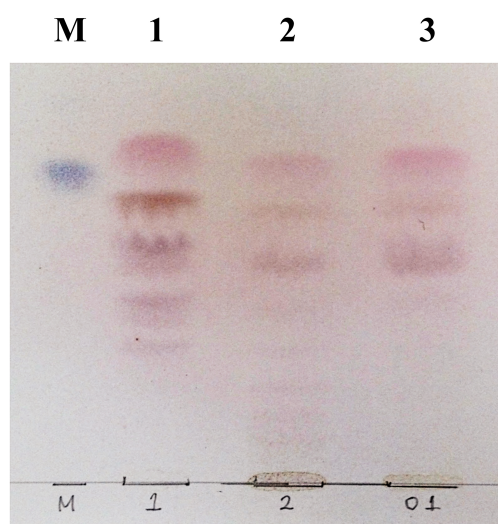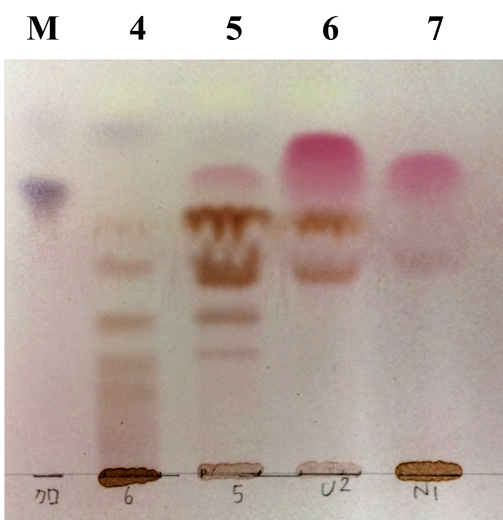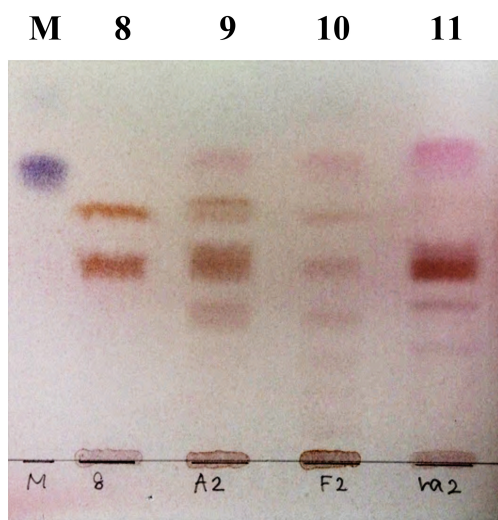

Supplement: S1 Fig — Description of lanes is provided in Fig 1 of the main text. (PDF) [file pone.0155537.s001.pdf]

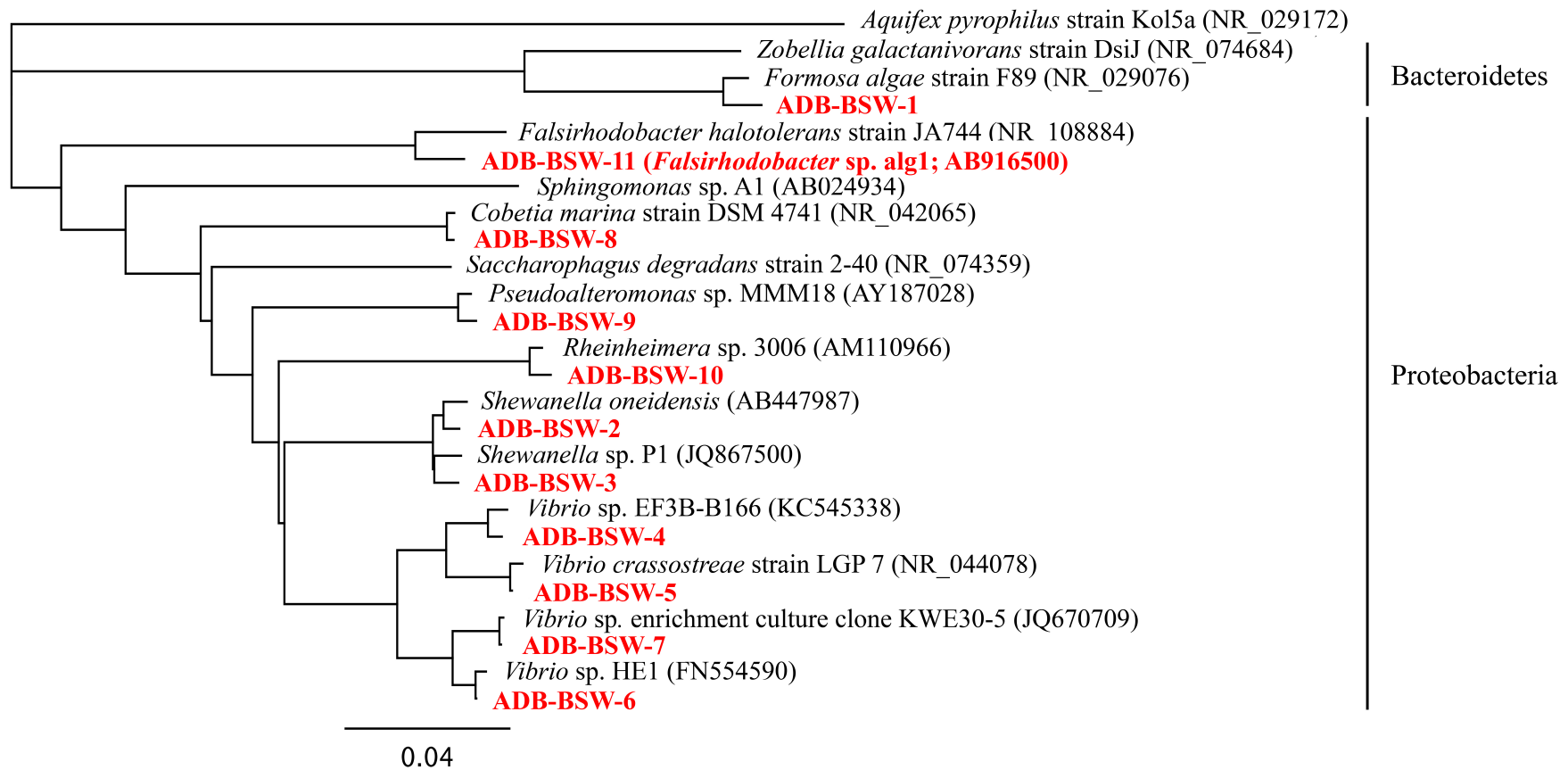

Supplement: S2 Fig — Isolates are indicated in red. Key alginolytic strains Zobellia galactanivorans strain DsiJ, Sphingomonas sp. A1 and Saccharophagus degradans strain 2–40 were used as reference. Aquifex pyrophilus strain Kol5a was used as the outgroup. Phylogenetic tree was generated using Geneious R8 v. 8.1.5. (PDF) [file pone.0155537.s002.pdf]

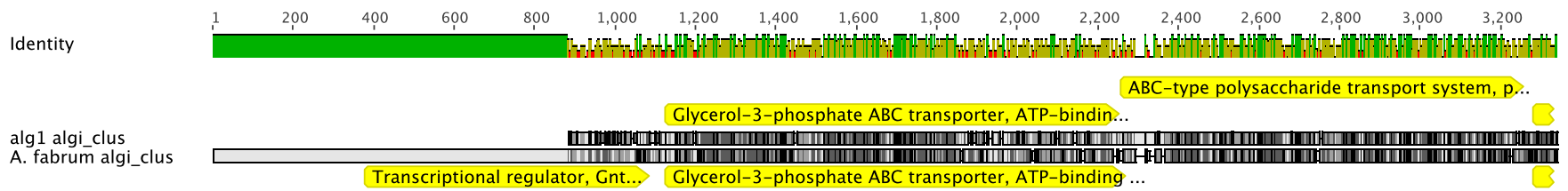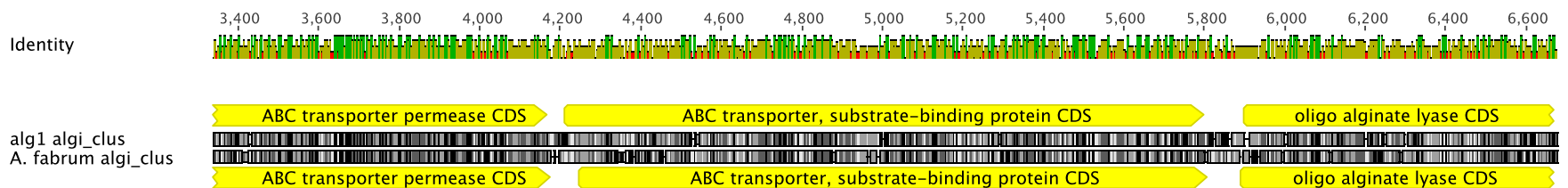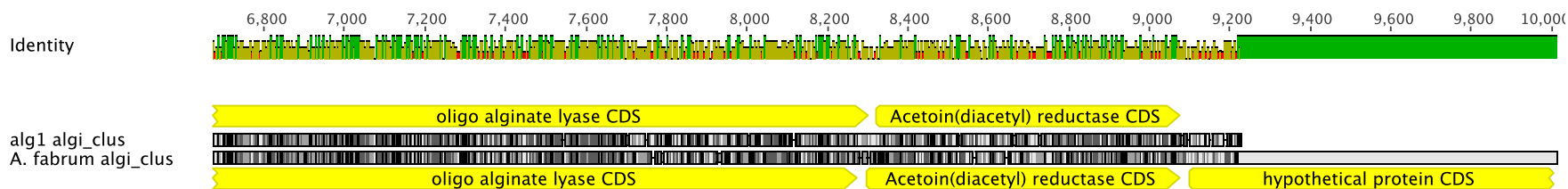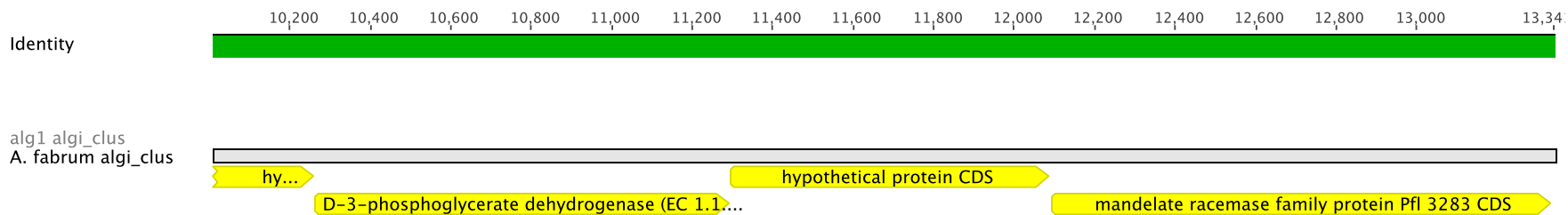

Supplement: S5 Fig — Multiple sequence alignment was generated using Geneious R8 v. 8.1.5. (PDF) [file pone.0155537.s005.pdf]
