# Supplementary material for: Falsirhodobacter sp. alg1 Harbors Single Homologs of Endo and Exo-Type Alginate Lyases Efficient for Alginate Depolymerization
Source: PLoS One. 2016 May 13;11(5):e0155537. doi: 10.1371/journal.pone.0155537 (PMC4866713; doi:10.1371/journal.pone.0155537)
Supplement: S3 Fig — Samples were separated in a 12% Mini-PROTEAN TGX Stain-Free Protein Gel (Biorad) and visualized with a standard UV transilluminator. (PDF) [file pone.0155537.s003.pdf]

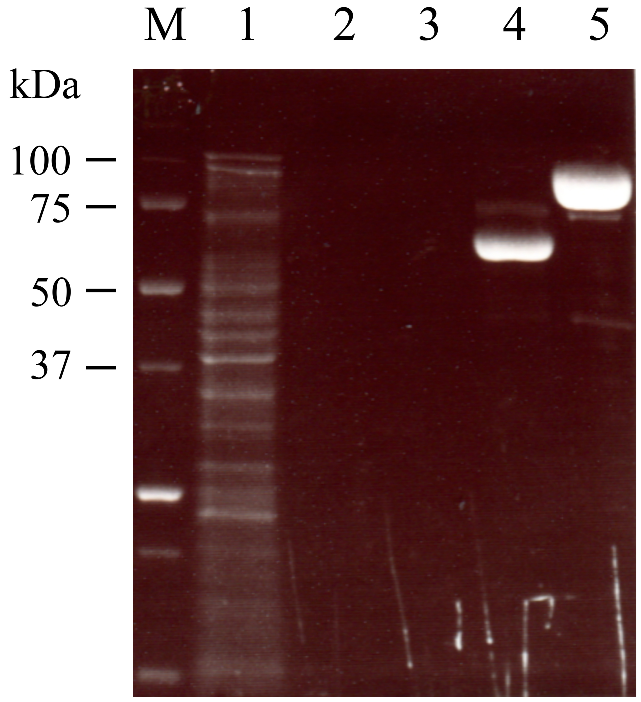

M: Precision Plus Protein Standard, 1: Flow through after wash, 2: Flow through after binding, 3: Flow through after elution, 4: Purified AlyFRA (estimated size 53.3 kDa), 5: Purified AlyFRB (estimated size 92.9 kDa). Only flow throughs for AlyFRA are shown.
