# Supplementary material for: Falsirhodobacter sp. alg1 Harbors Single Homologs of Endo and Exo-Type Alginate Lyases Efficient for Alginate Depolymerization
Source: PLoS One. 2016 May 13;11(5):e0155537. doi: 10.1371/journal.pone.0155537 (PMC4866713; doi:10.1371/journal.pone.0155537)
Supplement: S4 Fig — Enzymatic activity of AlyFRB in response to a. cationic ions, b. temperature and c. alginate depolymerization time. (PDF) [file pone.0155537.s004.pdf]

a. **M 1 2 3 4**

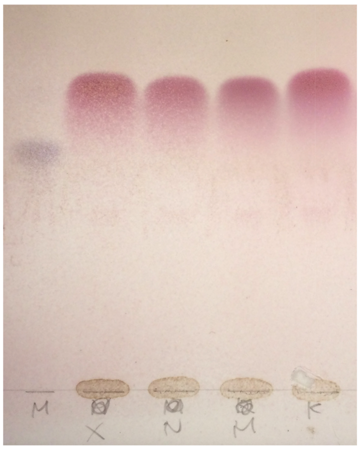

M: Hydrolyzed sodium alginate, 1: MOPS, 2: NaCl, 3: $MgCl_2$, 4: KCl

b. **M 1 2 3 4 5**

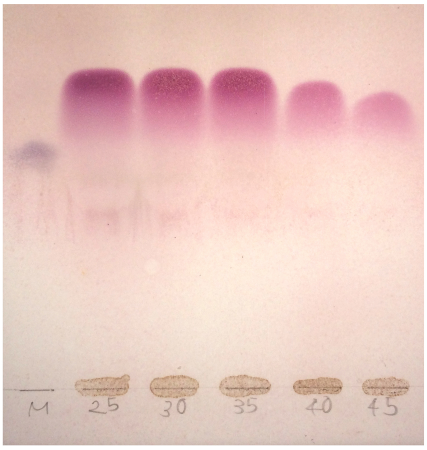

M: Hydrolyzed sodium alginate, 1: 25℃, 2: 30℃, 3: 35℃, 4: 40℃,5: 45℃

c. **Time (h): 0 1 2 4 8 16**

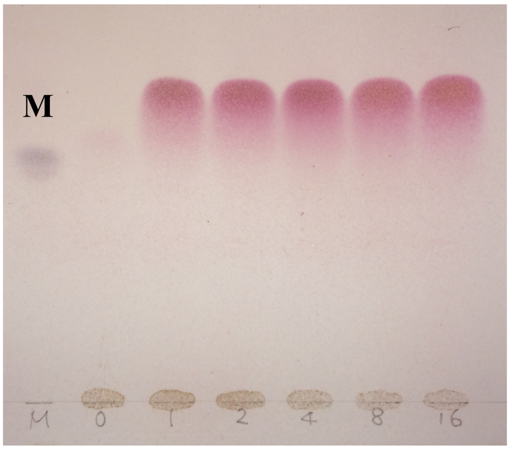

M: Hydrolyzed sodium alginate
